# Enteric Ganglioneuritis, a Common Feature in a Subcutaneous TBEV Murine Infection Model

**DOI:** 10.3390/microorganisms9040875

**Published:** 2021-04-18

**Authors:** Mathias Boelke, Christina Puff, Kathrin Becker, Fanny Hellhammer, Frederic Gusmag, Hannah Marks, Katrin Liebig, Karin Stiasny, Gerhard Dobler, Wolfgang Baumgärtner, Claudia Schulz, Stefanie C. Becker

**Affiliations:** 1 Department of Infectious Diseases, Institute for Parasitology, University of Veterinary Medicine, Buenteweg 17, 30559 Hanover, Germany; mathias.boelke@tiho-hannover.de (M.B.); fanny.hellhammer@tiho-hannover.de (F.H.); frederic.gusmag@tiho-hannover.de (F.G.); katrin.liebig55@gmail.com (K.L.); 2 Research Center for Emerging Infections and Zoonoses, Buenteweg 17, 30559 Hanover, Germany; Claudia.schulz@tiho-hannover.de; 3 Department of Pathology, University of Veterinary Medicine, Buenteweg 17, 30559 Hanover, Germany; christina.puff@tiho-hannover.de (C.P.); kathrin.becker@tiho-hannover.de (K.B.); Hannah.marks@tiho-hannover.de (H.M.); wolfgang.baumgaertner@tiho-hannover.de (W.B.); 4 Department of Virology, Medical University of Vienna, Kinderspitalgasse 15, 1090 Vienna, Austria; karin.stiasny@meduniwien.ac.at; 5 Institute of Microbiology of the Bundeswehr, Neuherbergstraße 11, 80937 Munich, Germany; gerharddobler@msn.com

**Keywords:** tick-borne encephalitis virus, ganglioneuritis, enteric nervous system, T lymphocytes

## Abstract

Tick-borne encephalitis (TBE) is a severe neurologic disease in Europe and Asia. Disease expression ranges from asymptomatic to severe neurological clinical pictures, involving meningitis, encephalitis, meningoencephalitis and potentially fatal outcome. Humans mostly become infected with TBE virus (TBEV) by the bite of an infected tick. Gastrointestinal (GI) symptoms in humans are mainly attributed to the first viremic phase of TBEV infection with unspecific symptoms and/or resulting from severe neurological impairment of the central nervous system (CNS). We used the subcutaneous TBEV-infection of C57BL/6 mice as a model to analyze GI complications of TBE. We observed the acute distension and segmental dilation of the intestinal tract in 10 of 22 subcutaneously infected mice. Histological analysis revealed an intramural enteric ganglioneuritis in the myenteric and submucosal plexus of the small and large intestine. The numbers of infiltrating macrophages and CD3^+^ T lymphocytes correlated with the severity of ganglioneuritis, indicating an immune-mediated pathogenesis due to TBEV-infection of the enteric plexus. Our study demonstrates that the inflammation of enteric intramural ganglia presents to be a common feature in TBEV-infected mice. Accordingly, the results of this mouse model emphasize that GI disease manifestation and consequences for long-term sequelae should not be neglected for TBEV-infections in humans and require further investigation.

## 1. Introduction

Tick-borne encephalitis (TBE) is a serious neurological disease in Eurasia, representing an increasing public health concern. In humans, TBE ranges from asymptomatic or mild forms to severe neurological disease manifesting as meningitis, encephalitis, meningoencephalitis and meningoencephalomyelitis, resulting in long-term sequelae and potentially fatal outcome [1,2,3]. The causative agent, TBE virus (TBEV) belongs to the genus Flavivirus (family: *Flaviviridae*), which further comprises several neurotropic viruses such as West Nile virus (WNV), Japanese encephalitis virus (JEV), Zika virus (ZIKV) or Powassan virus (POWV). It is believed that the case fatality rates differ between the three main TBEV subtypes, ranging from 1%–2% for the European, 6%–8% for the Siberian to 20%–30% for the far-eastern subtype [3,4]. Recently, two new TBEV subtypes have been described: the Himalayan and the Baikalian [5,6]. In addition, TBEV has newly emerged in regions previously proclaimed as non-endemic, with recent findings of TBEV in the United Kingdom and to higher altitudes in Austrian alpine regions [7,8,9,10] and Tunisia representing the first ever report of TBEV in an African country [11]. Mostly, people get infected with TBEV via the bite of an infected tick. The virus subsequently infects dermal dendritic cells at the site of the tick bite, as shown by the detection of viral antigen in Langerhans cells. Following initial infection at the skin side, the virus may be transported to the lymph nodes, as indicated by the migration of infected Langerhans cells [12]. After an incubation period of 7–10 days, unspecific general symptoms similar to flu occur in 70% of the cases (first clinical phase or viremic phase) [13]. A second clinical phase with central nervous system involvement occurs in 20%–30% of the cases, appearing as meningitis (50%), meningoencephalitis (40%) and meningoencephalomyelitis (10%) [14].

While central nervous system (CNS) symptomatology is mostly described in TBEV infection, gastrointestinal (GI) symptoms in humans are sporadically reported. Those non-specific symptoms often occur during the first clinical phase before the onset of neurologic symptoms or as sequelae resulting from neurological damage. The frequency of GI autonomic dysfunction in TBEV infection was studied in a prospective study including 656 German TBE patients between 1994 and 1998 [13]. An impaired bowel function was reported in 1% of patients presenting with meningoencephalitis and in 13.5% of patients presenting with meningoencephalomyelitis. Furthermore, another human case study reported three TBE patients showing autonomic dysfunction in the upper and/or lower GI tract including severe postprandial abdominal pain and vomiting, reduced bowel motility and constipation [15]. In addition, a case of a 75-year-old man showing severe impairment of intestinal propulsive motility mimicking bowel obstruction after a TBEV infection involving an encephalomyeloradiculitis, was reported [16]. The authors hypothesized a potential myenteric plexus infection, but no molecular pathologic confirmation of virus presence in myenteric cells was conducted. A TBEV strain of low pathogenicity was isolated from an area with a human case with mainly GI symptoms and weight loss [17]. Pseudo-obstruction of the colon after a tick bite was firstly described in 1989, but without confirmation of a TBEV infection [18]. Although this should be self-evident, descriptions of GI symptoms in alimentary TBEV infections are very rare due to the limited number of oral infections and the resulting lack of data. However, Gritsun et al. [1] mention symptoms such as nausea and vomiting, similar to the first phase of infection upon tick bite. As the recovery rate without neurological sequelae is quite high in comparison to tick-bite-induced TBE, potential GI symptoms due to damage of the CNS are not described.

First attempts to investigate TBEV-infection associated GI symptoms in more depth to understand underlying mechanisms have been made in the mouse model. The experimental infection of mice via the intravenous route showed the infection of enteric neurons with TBEV, resulting in intestinal distension caused by peripheral neuritis and paresis, leading to death [19]. Immunohistochemical analyses revealed the presence of viral antigens in the enteric and celiac plexus. Further, GI symptoms after subcutaneous infection with other flaviviruses such as WNV and ZIKV have been described in mice [20,21]. White et al. [21] analyzed the impact of subcutaneous flaviviral infection (WNV, ZIKV and POWV) on the motility of the GI tract in a mouse model using C57BL/6 mice. They observed a delayed GI transit upon infection caused by damage to enteric neurons manifesting as GI motility abnormalities. Furthermore, the acute dilation of the small and large intestine was described and viral antigen was detected localizing to the enteric ganglia.

Intradermal and subcutaneous infections with TBEV are considered a reproducible method of a natural human infection following the bite of an infected tick. Accordingly, we used this model to analyze GI symptoms in a more natural infection model using the European TBEV prototype strain Neudoerfl in the immunocompetent C57BL/6JOlaHsd mouse strain. Both mice and virus strain were chosen because they constitute well-characterized models and to increase the comparability of our study with other studies on flavivirus infections in models systems [21].

## 2. Materials and Methods

### 2.1. Ethical Statement

All animal experiments were performed in compliance with the German Animal Welfare Law (TierSchG BGBl. S. 1105; 25.05.1998). The mice were housed and handled in accordance with good animal practice as defined by FELASA. All animal experiments were approved by the responsible state office (Lower Saxony State Office of Consumer Protection and Food Safety) under permit number AZ 33.9-42502-04-18/2804.

### 2.2. Mice

The 5-week-old female C57BL/6JOlaHsd mice were purchased from a commercial breeder (Envigo, Rossdorf, Germany). The mice were housed in an IsoCage N Biocontainment system with individually ventilated cages (Tecniplast, Hohenpeißenberg, Germany) in groups of 2–4 animals per cage with one-week acclimatization before the start of experiments. Animal housing and handling took place under Biosafety level 3** (BSL3**) conditions in a 12 h light/12 h dark light regime. A sterilized pellet diet and water were supplied *ad libitum*.

### 2.3. Virus and Cell Culture

TBEV-European (TBEV-Eu) prototype strain Neudoerfl (GenBank accession number U27495) was obtained from the collection of the Department of Microbiology of the German Armed Forces in Munich, Germany. Virus was propagated in A549 cells (ATCC^®^ CCL-185™, American Type Culture Collection, Manassas, Virginia, USA) in minimal essential medium (MEM) with 2% fetal bovine serum (FBS) and antibiotics (penicillin/streptomycin PanBiotech; Aidenbach, Germany, gentamicin/amphotericin Thermo Fisher, Waltham, MA, USA) for one passage and viral titer was determined by serial dilution and the calculation of the tissue culture infectious dose 50% (TCID50/mL) according to Reed and Muench [22]. All experiments were performed under BSL3** conditions.

### 2.4. Experimental Design

After one week of acclimatization, 22 mice (M7-M28) were subcutaneously infected into the neck with 100 µL containing 1000 PFU of TBEV strain Neudoerfl. Three mice served as negative controls and received 100 µL of MEM (Thermo Fisher, Waltham, MA, USA) without supplements subcutaneously. As positive controls, two animals (M29-M30) were infected intracerebrally with 20 µL containing 200 PFU of TBEV Neudoerfl. The site of intracerebral injection is halfway between eye and ear, just off the midline and the needle injection depth was 4 mm to avoid extending too deeply into the brain [23]. For evaluation of the alimentary infection, in addition, 22 mice (M7-M28) were infected orally with 25 µL of MEM (Thermo Fisher, Waltham, MA, USA) containing 1000 PFU of TBEV strain Neudoerfl. Additionally, three mice served as negative control and received 25 µL of MEM (Thermo Fisher, Waltham, MA, USA). Mice were checked daily for body weight and clinical score. The clinical score included bodyweight, cardiovascular condition, coat/skin condition, respiratory tract symptoms, the environment, social behavior/general condition and locomotion as well as neurological scoring (see Figure S1 for the clinical score sheet). At pre-defined time points (0, 2, 4, 7, 10, 14, 17 and 21 days after infection (dpi)), three animals were euthanized under isoflurane anesthesia by decapitation. However, the clinical scores of subcutaneously infected animals made deviations from the defined time points necessary because humane endpoints [24] were reached earlier than planned. Therefore, mice from this infection were grouped according to disease progression into: (1) early phase group (2 dpi), (2) preclinical phase (no apparent sign of disease day 4 to 7), (3) clinical phase (apparent signs of disease leading to euthanasia of the respective animal, day 8 to 12), (4) asymptomatic (no sign of disease at any given time point of the infection past day 7) as well as (5) negative controls (no infection) for all further analysis. Serum and organs (cerebrum, cerebellum, spinal cord, liver, spleen, intestine, lymph nodes) were collected for real-time quantitative reverse transcription PCR (RT-qPCR; see details below) and stored at −80 °C. Tissue samples for histological analysis were fixed with 10% formalin for a minimum of 48 h before further processing to inactivate potential infectious virus followed by embedding in paraffin wax (FFPE).

### 2.5. RNA Extraction

Tissue samples were homogenized with steel beads in 500 µL of MEM with antibiotics (penicillin/streptomycin PanBiotech; Aidenbach, Germany, gentamicin/amphotericin Thermo Fisher, Waltham, MA, USA) using a TissueLyser II (Qiagen, Hilden, Germany) with 20 Hz for 1 min. Homogenates were clarified by centrifugation. Serum samples were pre-diluted 1:10 in cell culture medium. Total RNA was extracted from 140 µL of serum or homogenate after heat inactivation in AVL/AVE buffer at 70 °C for 20 min using the QAIamp Viral RNA Mini QiaCube kit (Qiagen, Hilden, Germany) at the QIAcube (Qiagen, Hilden, Germany) following the manufacturer’s instructions. Until further processing, samples were stored at −80 °C.

### 2.6. Real-Time Quantitative RT-PCR

Samples were screened by duplex real-time quantitative reverse transcription-PCR (RT-qPCR) for TBEV-RNA [25] and β-actin-RNA copies [26] following a modified protocol developed by Schwaiger and Cassinotti. TBEV RNA copies were determined using a standard curve. Therefore, TBEV RNA from the Austrian Neudoerfl strain (U27495.1) was used in a tenfold serial dilution, while RNase-free water served as the negative control. Samples were run in duplicates using the Qiagen One-Step RT-PCR Kit (Qiagen, Hilden, Germany), the AriaMx Real-time PCR System (Agilent Technologies, Santa Clara, California, USA) and results were analyzed using the AriaMx software version 1.5 (Agilent Technologies, Santa Clara, CA, USA).

### 2.7. Histology and Immunohistochemistry

For histological analysis, FFPE-embedded samples were cut at 2–3 µm and stained with haematoxylin and eosin (H&E) according to standard protocols.

Immunohistochemistry was performed using the avidine–biotin–peroxidase complex method with antibodies specific for CD3 for T lymphocytes, CD45R for B lymphocytes and CD107b for macrophages (Table 1). Furthermore, virus detection was performed with a polyclonal antibody directed against TBEV (Table 1). Immunohistochemistry was carried out as described before using 3,3′-diaminobenzidine tetrahydrochloride as a chromogene [27,28].

Positive controls included murine lymphoid tissue for CD3, CD45R and CD107b and nervous tissue from a spontaneously TBEV-infected dog for TBEV, respectively. For negative controls, primary antibodies were replaced by serum from non-immunized rats (for monoclonal antibodies) and non-immunized rabbits (for polyclonal antibodies).

The immunohistochemical evaluation of the small and large intestine was performed by counting marker-expressing cells within the intramural ganglia and muscle layer. Furthermore, the area of the muscle layer was determined morphometrically to allow the calculation of positive cells per µm^2^.

### 2.8. Statistical Analysis

Statistical analysis was conducted on the previously defined groups 1–5 using GraphPad Prism V8.3.1 (San Diego, CA, USA). Statistical differences in infiltrating CD3^+^ T cells, Mac3+ macrophages and TBEV antigen-positive cells were analyzed for groups 1–3 by using the Kruskal–Wallis test (*p* < 0.05) and individual groups were compared by using a Mann–Whitney-U-test with Bonferroni-corrected p values (*p* < 0.02). To evaluate the dependency of the occurrence of ganglioneuritis with detection of viral antigen, a McNemar analysis was conducted using GraphPad Prism V8.3.1 (San Diego, CA, USA).

## 3. Results

### 3.1. Course of Infection

To further increase our understanding of GI infection in a subcutaneous infection TBEV mouse model, 22 mice were infected with 1000 viral particles of the TBEV strain Neudoerfl into the neck. This infection method is discussed to better simulate natural tick-bite infection than the intraperitoneal and intramuscular application of tick-borne viruses [29]. Three mice served as day 0 controls, three mice infected with cell culture medium served as negative controls and two mice were intracerebrally infected with 200 viral particles as positive controls (neurovirulence control).

Bodyweight loss as the first clinical symptom was observed at 7 dpi. Further clinical signs were observed after 8 dpi including a hunchback posture (11 mice), ruffed fur (11 mice), moribund condition (six mice), and hind leg paresis (five mice). Of all infected mice, except for one (M16), 9 of 11 mice (M18-26) showed severe clinical signs and reached the pre-defined humane endpoint for euthanasia between 8 and 12 dpi while two symptomatic mice (M17, M27) were euthanized before reaching humane endpoint. Due to the clinical score (Table 2, Figure S1), the day of euthanization might differ from the defined scheme. Viral RNA copies in the small intestine were first detected by RT-qPCR at 4 dpi, reaching highest levels at 7 and 8 dpi. In the central nervous system (cerebrum, cerebellum and spinal cord), TBEV RNA copies were found after 7 dpi with a peak in copy numbers at 10 dpi (Figure S2, Table S1).

Intracerebrally infected positive control animals reached the humane endpoint at 6 dpi. Both animals (M29 and M30) showed 10%–11% body weight loss, ruffed fur, hunchback posture, paresis of limbs, and moribund condition. The three mock-infected mice (M1-3) showed no obvious clinical signs and clinical score over course of the experiment.

Furthermore, we used oral infection to simulate an alimentary infection and to compare the GI symptoms with our subcutaneous infected mice. Therefore, 22 mice (Mo7–28) were fed with 1000 viral particles of the same TBEV strain and three animals served a negative control (Mo1-3) (Table S2). None of the infected animals developed clinical symptoms and we did not detect TBEV RNA in the intestine of those animals. However, we were able to detect viral RNA in the cerebrum of two animals (Mo 15 and 16) and in the cerebellum of three animals (Mo16, 19 and 20). Furthermore, one of the animals positive for viral RNA (Mo16) showed mild meningitis and one animal showed mild encephalitis (Mo20).

### 3.2. Macroscopic Alterations of the GI Tract

**Figure 1:**
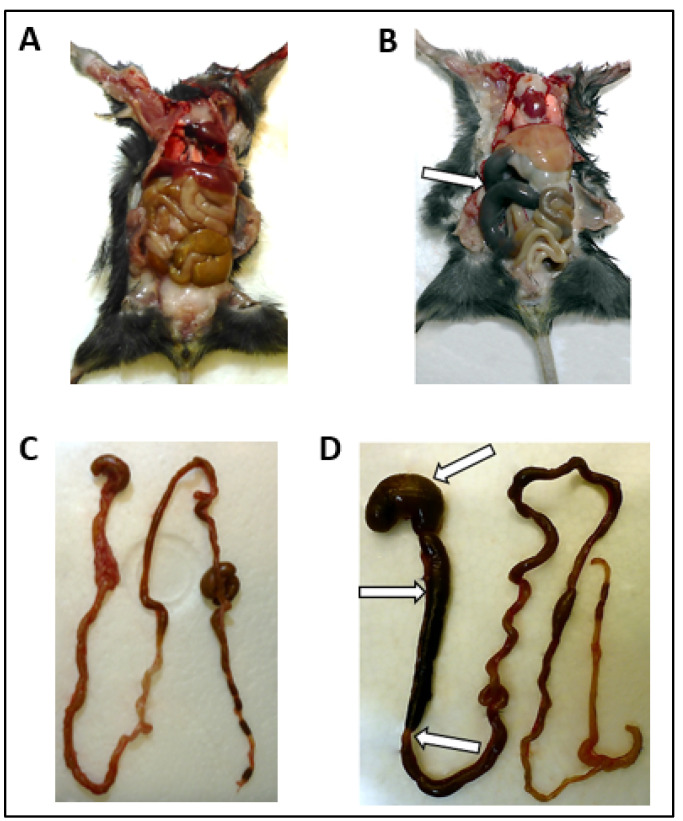
Macroscopic findings of the GI tract from subcutaneously TBEV-infected mice ((**B**) M26 at 8 dpi; (**D**) M22 at 10 dpi) and mock-infected control M1 at 14 dpi (**A**,**C**). Segmental distension and alterations of the small intestine and the stomach are indicated by white arrows. Acute GI tract alterations were found in subcutaneously TBEV-infected mice after 8 dpi.

Macroscopic alterations of the GI tract occurred after 8 dpi and included considerable segmental dilation and distension of the small intestine and stomach (Figure 1B,D) compared to the GI dimensions of the mock-infected mice (Figure 1A,C). Arrows indicate regions of segmental dilation as well as distension and transition to normal-appearing regions. Overview of presence and localization of macroscopic GI alterations is given in Table 2. The proximal (nine mice) and the mid part (eight mice) of the small intestine were mostly affected, while the stomach (Figure 1D) was dilated in five mice and the distal part of the small intestine showed alterations in only one mouse (Table 2). None of the positive and mock-infected controls, as well as none of the orally infected animals showed evidence of bowel dilation.

### 3.3. Ganglioneuritis in Intramural Ganglions of Small and Large Intestine and Viral Antigen in Enteric Plexus

Next, we analyzed the underlying cause of the macroscopic GI tract alterations. Sections of the small and large intestine were stained with hematoxylin and eosin and antibodies detecting viral antigen.

**Figure 2:**
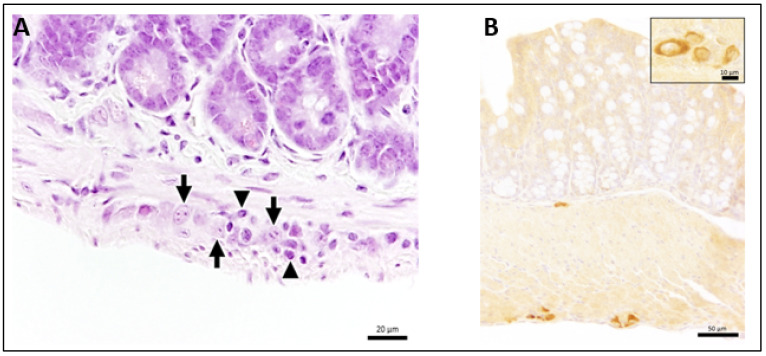
(**A**) H&E staining showing ganglioneuritis of the myenteric plexus of M21 at 11 dpi. Neurons of the ganglion (arrows) are surrounded by inflammatory cells (arrowheads). (**B**) Staining for TBEV antigen in the large intestine of M18 at 9 dpi. TBEV antigen is present in the myenteric and submucosal plexus of the large intestine. The insert shows the intracytoplasmic localization of viral antigen. Staining 1:800 with anti-TBEV-rabbit serum. Bars represent 20 µm (**A**) and 50 µm as well as 10 µm for the insert (**B**).

Hematoxylin and eosin staining revealed intramural ganglioneuritis with infiltrating inflammatory cells of the myenteric and submucosal plexus in the small and/or large intestine in 15 subcutaneously infected mice (Figure 2A). Ganglioneuritis was observed in one animal at day 2 and one animal at day 4 (Table 2). At day 7, two mice showed ganglioneuritis and subsequently, all TBEV-infected mice presented with ganglioneuritis of varying severity (Table 2). In addition, two mice without macroscopic alterations (M16 and M21 and the two intracerebrally infected controls were positive for ganglioneuritis. Ganglioneuritis was found in the myenteric plexus (Auerbach plexus) and the submucosal plexus (Meissner plexus). Staining with anti-TBEV-rabbit serum (Table 1) revealed intracytoplasmic viral antigen in the enteric plexus of subcutaneously TBEV-infected mice first at day 7 (Figure 2B) in M15 and was subsequently found in seven other mice (M16–18, 22, 25–27). One intracerebrally infected animal displayed virus in the enteric plexus at day 6. The occurrence of ganglioneuritis significantly associated with presence of viral antigen (*p* = 0.0133) as evaluated by McNemar analysis.

### 3.4. Numbers of CD3-Positive Cells and Macrophages Were Increased in Mice with Ganglioneuritis

**Figure 3:**
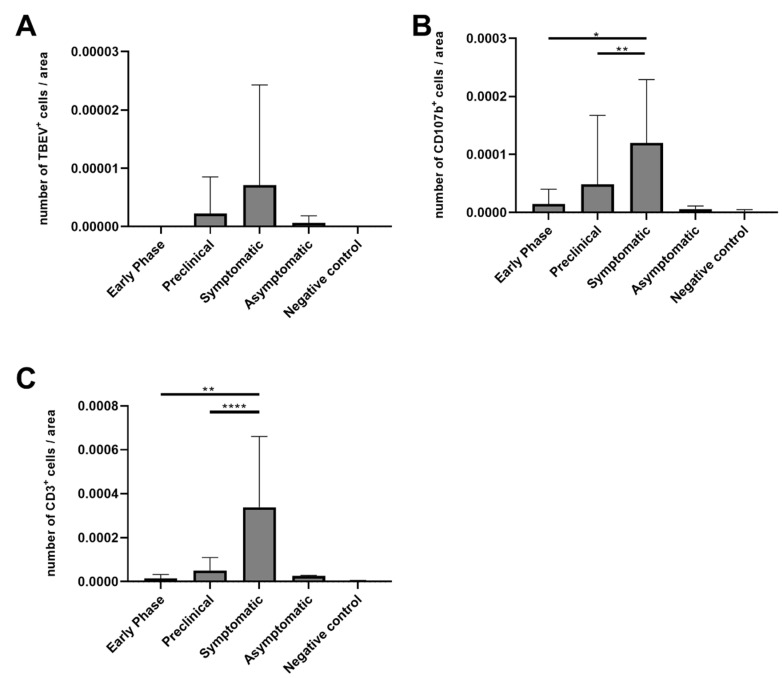
Relative number of specific cell types over the course of infection compared to the total area of the muscularis (m^2^). (**A**) TBEV^+^ cells, (**B**) CD107b^+^ cells, (**C**) CD3^+^ cells. Significant differences between each of the three groups (early phase, preclinical and symptomatic) detected by Mann–Whitney-U-test with Bonferroni corrected *p* values using GraphPad Prism software are indicated by asterisks (*p 0.033, ** *p* 0.0033 and **** *p* 0.000033).

The analysis of prominent cells during ganglioneuritis showed only marginal numbers of CD45^+^ cells, therefore we did not count CD45^+^ cells for all slices. The results for virus-positive cells, CD107b^+^ (macrophages) and CD3^+^ (T lymphocytes) cells are shown in Figure 3A–C. TBEV-positive cells were found in the tissue of animals during the clinical disease phase (8–12 dpi) but in some animals they were also apparent in the preclinical phase (M15) as well as in asymptomatic animals (M16) (Figure 3A), whereas negative control animals and animals that were euthanized without obvious clinical signs in the preclinical phase did not yield any TBEV-positive cells. Moreover, no significant difference between the early phase, the preclinical group and symptomatic group could be detected. Numbers of CD107b^+^ cells per area were significantly higher in the symptomatic phase compared to the early (*p* = 0.0085) and the preclinical phase (*p* = 0.0023) (Figure 3B). CD3^+^ T cells were the most abundant inflammatory cell type observed in the GI tract and the highest numbers correlated with presence of macroscopic GI tract alterations and the presence of ganglioneuritis. Significantly higher numbers were observed in the symptomatic phase compared to the early (*p* = 0.0027) and preclinical phase (*p* < 0.0001) (Figure 3C). Furthermore, comparing all the three groups (early phase, preclinical and symptomatic phase) by use of the Kruskal–Wallis test revealed no significances for TBEV+ cells while significances were found for CD107b^+^ (*p* = 0.0024) and CD3^+^ (*p* = 0.0001) cells.

## 4. Discussion

To analyze the phenomenon of GI dysfunction after TBEV infection, we used the subcutaneous route of TBEV infection, simulating a tick bite. We chose to use C57BL/6J-OlaHsd mice to increase the comparability with the study of White et al. on GI dysfunction after subcutaneous flavivirus infection [21]. Furthermore, C57BL/6JOlaHsd mice were shown to be more susceptible to TBEV [12]; however, these mice are also less suitable for tick infestation, limiting our model to the subcutaneous injection. In this regard, it has to be considered that subcutaneous infection may be closer to a tick-bite infection than intraperitoneal infection [29] but still lacks some characteristics of a tick infection, such as tick saliva and the duration of virus application, which can change the course of infection compared to a natural TBEV infection. Specifically, tick saliva has been shown to enhance infection TBEV replication in dendritic cells [30] which can have a severe impact on viremia and disease progression. However, as a first approach towards a simulation of tick-bite infection, we used the well-established subcutaneous infection model. After the infection of C57BL/6JOlaHsd mice with the European prototype TBEV strain Neudoerfl, we observed abdominal distension, alterations of the GI tract and segmental dilation of the small intestine acutely appearing in all animals except one mouse after 8 dpi. Similar to mice intravenously infected with the highly pathogenic far eastern TBEV Sofjin strain [19], clinical signs were severe after subcutaneous infection with the TBEV-EU Neudoerfl strain and humane endpoints were reached between 8 and 12 dpi in 9 of 11 symptomatic animals in our study. In contrast, none of the orally infected animals showed any clinical signs. However, we were able to show TBEV infection in four out of 22 infected animals and two animals showed pathological alterations in the CNS with mild meningitis (Mo16) and mild encephalitis (Mo20, Table S2). These data show that we induced TBEV infection in a small proportion of the animals but the virus dose of 1000 viral particles per animal was too low to efficiently induce infection via the alimentary route. This observation is in line with a study analyzing the TBEV infection in natural rodent hosts *Apodemus agrarius* where 100–1500 viral particles were used for infection and subclinical viral encephalitis was observed [31].

Besides TBEV, other neurotrophic flaviviruses were also shown to cause GI dysfunction in mice, suggesting a common mode of infection of different flaviviruses in the subcutaneous infection mouse model. Besides macroscopic alterations, an infiltration of CD3^+^ lymphocytes in the myenteric plexus was observed in all flaviviral subcutaneous infections [21] including TBEV in our study. Thus, a systemic infection with neurotropic viruses could lead to immune-mediated damage of the enteric nervous system, leading to an intestinal dysmotility disorder in mice, highlighting the model features of acute intestinal pseudo-obstruction in human chronic irritable bowel syndrome (IBS) [21,32]. A similar phenomenon might be responsible for the GI tract alteration found in our study. To further analyze the involvement of immune–pathological processes in symptom development in our infection model, we used immunohistochemical antibody staining of TBEV antigen and cell markers, as well as RT- qPCR for TBEV. The immunohistochemical staining revealed intramural enteric ganglioneuritis in 13 mice from 7 dpi onwards (M15-M28) affecting the myenteric (Auerbach’s) plexus and the submucosal (Meissner´s) plexus of the small and large intestine. Both are incorporated in the enteric nervous system (ENS), which is comprised of glial and complex neural networks. The myenteric plexus, which is situated between inner circular and outer longitudinal muscle layers of the bowel (muscularis propria) regulates mainly the motility and peristaltic of the GI tract, primarily independent from the CNS, and acts as major nerve supply [33,34,35]. The submucosal plexus is located between the muscularis propria and the mucosa, regulating intestinal blood flow, intestinal epithelial function and repair as well as responses to sensory stimuli [34]. Therefore, the ganglioneuritis of glial cells may have a substantial impact on the motility and peristaltic of the GI tract [35]. Again, this is in line with the study of White et al. that showed that infection and damage of the enteric nervous system with flaviviruses result in an intestinal dysmotility syndrome with delayed GI transit [21].

In our study, all samples of the subcutaneous infection series tested positive for TBEV RNA (Supplementary Table S1, Supplementary Figure S1). Ganglioneuritis was significantly (*p* = 0.0133) associated with the detection of TBEV-antigen as viral antigen was detected in eight of the 15 animals with ganglioneuritis. TBEV-antigen was firstly detected in the myenteric plexus at 7 dpi in the small and large intestine in a mouse with mild ganglioneuritis. Similarly, in intravenously TBEV-infected mice, viral antigen in enteric ganglia was first detected at 5 dpi and subsequently in all examined animals at 7 dpi [19] and for WNV-infected mice at 6 dpi [21]. In contrast, one animal (M18) in our study showed high amounts of viral antigen in the large intestine, while no evidence of ganglioneuritis could be observed despite the presence of ganglioneuritis in the small intestine. A possible explanation for the differences in the sensitivity to detect TBEV with any of the two methods could be that viral antigen loads might have been too low for a reliable immunohistochemical antibody detection in the respective negative samples, while the diagnostic sensitivity of RT-qPCR to detect TBEV-RNA is high (detection limit of 1–10 copies/µL). On the other hand, viral RNA detection via RT-qPCR does not exclude a contamination of the tissue by viral RNA containing blood or feces in case of the large and small intestine.

Interestingly, in our study enteric ganglioneuritis was also observed in both intra-cerebrally infected positive control mice as well as in one mouse (M16) without clinical signs or viral RNA, as determined by RT-qPCR analysis. However, in the latter animal, small amounts of TBEV antigen were detected in the small intestine. The presence of a ganglioneuritis and viral antigen in the small intestine in intracerebrally infected mice is remarkable and might give arguments against the hypothesis that TBEV invades the CNS via the autonomic nerves running from the enteric plexus. Furthermore, we were able to detect TBEV RNA in the brain but not in the intestine of some of the orally infected animals. Alternatively, virus- or immune cell-induced damage of regions in the brainstem and medulla oblongata may lead to the neuronal inhibition of axons to the GI tract. Interestingly, the brainstem and medulla oblongata are considered as two major regions of TBEV-induced lesions in the CNS [36]. Those lesions could explain the often-seen GI sequelae following neurologic TBEV infections in humans, but this hypothesis requires further investigation.

In line with observation in the WNV infection model, the presence of ganglioneuritis and degree of severity were linked to amounts of infiltrating macrophages and CD3^+^ T lymphocytes in our study. Significantly higher amounts of macrophages and CD3^+^ T cells were found in the symptomatic phase in comparison to the preclinical and early phase of infection (Figure 3). CD3^+^ T cells recruitment in turn will influence the activation of CD8^+^ cells. The role of CD8^+^ cells in flavivirus infection has already been demonstrated by several studies. For instance, infiltrating CD8^+^ T lymphocytes were described to be primarily responsible for the early GI tract alterations in mice infected with WNV as CD8^+^-deficient mice did not develop GI tract alterations and the adoptive transfer of CD8^+^ cells resulted in observed alterations for immunocompetent mice [21]. Regarding the role of CD8^+^ cells in TBEV infection, systemic inflammatory (e.g., CD8^+^ cell-mediated) and stress responses, in addition to CNS disease, contributed to the fatal outcome of infection, highlighting the effect of immune-induced damages [37,38]. Furthermore, fatal human TBE cases were linked to activated CD8^+^ T cells [36,39]. The humoral response did not appear to have a significant role during ganglioneuritis, as CD45^+^ cells were just marginally found, matching findings in Langat virus (LGTV)-infected macaques’ brains, where only relatively low numbers of B cells infiltrated the CNS [40]. Similarly, CD45^+^ staining was marginal in our samples and thus not persuaded further.

## 5. Conclusions

To sum up, our study demonstrates the presence of acute GI alterations after subcutaneous TBEV infection caused by ganglioneuritis in the myenteric and submucosal plexus of small and large intestine. Significantly high amounts of infiltrating CD3^+^ T lymphocytes and, to a lesser amount, macrophages were observed in animals with ganglioneuritis and macroscopic alterations of the GI tract. Therefore, TBEV infection and subsequently, damage of neurons of the ENS by neurotropic flaviviruses, may be a source of acute and persistent GI pathology and dysmotility [21]. Accordingly, implications on life quality due to flavivirus-induced GI complications and sequelae should not be neglected. Furthermore, the possibility of oral TBEV infection in humans suggests that further investigations of the TBEV-induced pathogenesis of the GI tract for both the tick-borne and food-borne infection routes are required.

## Figures and Tables

**Table 1:** Antibodies and related reagents used for immunohistochemical staining.

1st Antibody				Pre-Treatment	2nd Antibody
Antigen, Target	Product Name	Clonality, Host Species, Clone	Dilution		
CD3, T lymphocytes	Dako A0452	Polyclonal, rabbit	1:200	Citrate buffer, microwave	Goat-anti-rabbit biotinylated
CD107b, microglia/macrophages	BioRad MCA2293B	Monoclonal, rat, clone M3/84	1:200	Citrate buffer, microwave	Rabbit-anti-rat biotinylated
CD45R, B lymphocytes	BD Bioscience 553085	Monoclonal rat, clone B220- biotinylated	1:1000	Citrate buffer, microwave	-
Anti-TBEV-rabbit serum	Center for Virology, Medical University of Vienna, Austria	Polyclonal, rabbit	1:800	Proteinase K	Goat-anti-rabbit biotinylated

All cell counts were analyzed in relation to the total area of the muscularis in analyzed sections.

**Table 2:** Overview of mice with enteral ganglioneuritis (15 of 22) after subcutaneous TBEV infection with the day of euthanasia after infection and clinical score (HEP= humane endpoint). The severity degree of ganglioneuritis ((+) = minimal, + = mild, ++ = mild to moderate, +++ = moderate, − = absent, n.d.= not detected), presence of viral antigen (+ = present, − = absent), presence of macroscopic alterations in GI tract regions (+ = present, − = absent) and status of viral RNA copies presence in intestine measured by qRT-PCR (+ = present, − = absent) are given for each animal.. M29 and M30 were intracerebrally infected positive control mice. No enteral ganglioneuritis, TBEV antigen, RNA or macroscopical alterations were found in the mock-infected negative control mice (data not shown).

Animal ID	dpi	Clinical Score	Ganglioneuritis	Viral Antigen	Macroscopic Alterations				qRT-PCR Intestine
					Stomach	Proximal	Mid	Distal	
**M8**	2	0	+	−	−	−	−	−	−
**M11**	4	0	+	−	−	−	−	−	(+)
**M15**	7	0	+	+	−	−	−	−	+
**M28**	7	0	(+)	−	−	−	−	−	+
**M24**	8	HEP	n.d.	n.d.	−	+	−	−	+
**M25**	8	HEP	+++	+	−	+	+	−	+
**M26**	8	HEP	++	+	+	+	(+)	−	+
**M18**	9	HEP	+	+	(+)	+	−	−	+
**M19**	9	HEP	++	−	−	+	+	−	+
**M17**	10	6	++	+	−	−	+	+	+
**M22**	10	HEP	++	+	+	+	(+)	−	+
**M27**	10	9	++	+	−	(+)	+	−	+
**M21**	11	HEP	+++	−	−	−	−	−	+
**M20**	12	HEP	+	−	−	+	+	−	+
**M23**	12	HEP	+	−	+	+	(+)	−	+
**M16**	14	0	+	+	−	−	−	−	−
**M29**	6	HEP	+	−	−	−	−	−	+
**M30**	6	HEP	+	+	−	−	−	−	+

## Data Availability

The data presented in this study are available on request from the corresponding author.

## Supplementary Materials

The following are available online at https://www.mdpi.com/article/10.3390/microorganisms9040875/s1. Table S1: overview of TBEV-RNA copies/500 µL homogenate (sample: size of a rice grain) as determined by RT-qPCR in the small intestine and cerebrum of mice subcutaneously infected with 103 TCID50/mL of TBEV Neudoerfl strain. M1-M3 were medium-injected negative controls while M29 and M30 were intracerebrally infected positive control mice. (dpi, day of euthanasia after TBEV-infection), Figure S1: clinical score sheet used for TBEV murine infection model, Figure S2: ratio (2delta-delta Cq) of Cq values of TBEV RNA copies/5 µL vs. β-actin copies /5 µL of small intestine, spinal cord, cerebrum, and cerebellum representing the relative expression differences over the course of infection. The spleen TBEV and β-actin RNA copies for each animal were used as reference organ, Table S2: overview of TBEV-RNA copies/500 µL homogenate (sample: the size of a rice grain) as determined by RT-qPCR in the small intestine, cerebrum, and cerebellum of mice orally infected with 103 TCID50/mL of TBEV Neudoerfl strain. M1-M3 were medium-injected negative control mice. (dpi, day of euthanasia after TBEV-infection).
