# Supplementary material for: Enteric Ganglioneuritis, a Common Feature in a Subcutaneous TBEV Murine Infection Model
Source: Microorganisms. 2021 Apr 18;9(4):875. doi: 10.3390/microorganisms9040875 (PMC8074024; doi:10.3390/microorganisms9040875)
Supplement: Supplementary file 1 [file microorganisms-09-00875-s001.zip › microorganisms-1176056-supplementary.pdf]

Supplementary Materials:

Tab. S1 Overview of TBEV-RNA copies/500 µl homogenate (sample: size of a rice grain) as determined by RT-qPCR in the small intestine and cerebrum of mice subcutaneously infected with 10^3 TCID50/ml of TBEV Neudoerfl strain. M1-M3 were medium injected negative controls while M29 and M30 were intracerebral infected positive control mice. (dpi, day of euthanasia after TBEV-infection).

Tab. S2: Overview of TBEV-RNA copies/500 µl homogenate (sample: size of a rice grain) as determined by RT-qPCR in the small intestine, cerebrum and cerebellum of mice orally infected with 10^3 TCID50/ml of TBEV Neudoerfl strain and histological findings within the brain. M1-M3 were medium injected negative control mice. (dpi, day of euthanasia after TBEV-infection).

Fig S1 Clinical Score sheet used for TBEV murine infection model.

Fig. S2 Ratio (2delta-delta Cq) of Cq values of TBEV RNA copies/5 µl vs β-actin copies /5 µl of small intestine, spinal cord, cerebrum and cerebellum representing the relative expression differences over the course of infection. The spleen TBEV and β-actin RNA copies for each animal were used as reference organ.

Tab. S1 Overview of TBEV-RNA copies/500 µl homogenate (sample: size of a rice grain) as determined by RT-qPCR in the small intestine and cerebrum of mice subcutaneously infected with 10^3 TCID50/ml of TBEV Neudoerfl strain. M1-M3 were medium injected negative controls while M29 and M30 were intracerebrally infected positive control mice. (dpi, day of euthanasia after TBEV-infection).

Animal ID	dpi	TBEV RNA copies in intestine	TBEV RNA copies in cerebrum
**M4**	0	Negative	Negative
**M5**	0	Negative	Negative
**M6**	0	Negative	Negative
**M7**	2	Negative	Negative
**M8**	2	Negative	Negative
**M9**	2	Negative	Negative
**M10**	4	$5.19 * 10^3$	Negative
**M11**	4	Negative	Negative
**M12**	4	$5.25 * 10^2$	Negative
**M14**	7	$1.06 * 10^4$	$6.17 * 10^4$
**M15**	7	$1.75* 10^7$	$4.63 * 10^6$
**M28**	7	$3.15 * 10^4$	$9.28 * 10^6$
**M24**	8	$1.52 * 10^6$	$1.59 * 10^9$
**M25**	8	$2.12 * 10^6$	$6.13 * 10^9$
**M26**	8	$1.23 * 10^6$	$2.71 * 10^8$
**M18**	9	$1.27 * 10^6$	$3.53 * 10^9$
**M19**	9	$1.00 * 10^6$	$1.74 * 10^9$
**M17**	10	$6.9 * 10^5$	$7.32 * 10^9$
**M22**	10	$7.08* 10^5$	$3.27 * 10^9$
**M27**	10	$9.22 * 10^5$	$6.89 * 10^8$
**M21**	11	$2.12 * 10^5$	$3.83 * 10^8$
**M20**	12	$3.65 * 10^5$	$1.08 * 10^9$
**M23**	12	$5.99 * 10^5$	$1.81* 10^9$
**M16**	14	Negative	Negative
**M1**	14	Negative	Negative
**M2**	14	Negative	Negative
**M3**	14	Negative	Negative
**M29**	6	$3.04 * 10^7$	$2.362 *10^{10}$
**M30**	6	$1.83 * 10^7$	$2,34 *10^{10}$

Tab. S2. Overview of TBEV-RNA copies/500 µl homogenate (sample: size of a rice grain) as determined by RT-qPCR in the small intestine, cerebrum and cerebellum of mice orally infected with 10^3 TCID50/ml of TBEV Neudoerfl strain and histological findings within the brain. M1-M3 were medium injected negative control mice. (dpi, day of euthanasia after TBEV-infection).

Animal ID	dpi	TBEV RNA copies in intestine	TBEV RNA copies in cerebrum	TBEV RNA copies in cerebellum	Histological findings within the brain
**Mo4**	0	Negative	Negative	Negative	Negative
**Mo5**	0	Negative	Negative	Negative	Negative
**Mo6**	0	Negative	Negative	Negative	Negative
**Mo7**	2	Negative	Negative	Negative	Negative
**Mo8**	2	Negative	Negative	Negative	Negative
**Mo9**	2	Negative	Negative	Negative	Negative
**Mo10**	4	Negative	Negative	Negative	Negative
**Mo11**	4	Negative	Negative	Negative	Negative
**Mo12**	4	Negative	Negative	Negative	Negative
**Mo13**	7	Negative	Negative	Negative	Negative
**Mo14**	7	Negative	Negative	Negative	Negative
**Mo15**	7	Negative	$6.37*10^3$	Negative	Negative
**Mo16**	10	Negative	$6.3*10^3$	$5.74*10^5$	Mild meningitis
**Mo17**	10	Negative	Negative	Negative	Negative
**Mo20**	10	Negative	Negative	$1.7*10^2$	Mild encephalitis
**Mo18**	14	Negative	Negative	Negative	Negative
**Mo19**	14	Negative	Negative	$4.68*10^4$	Negative
**Mo21**	14	Negative	Negative	Negative	Negative
**Mo22**	17	Negative	Negative	Negative	Negative
**Mo23**	17	Negative	Negative	Negative	Negative
**Mo24**	17	Negative	Negative	Negative	Negative
**Mo25**	21	Negative	Negative	Negative	Negative
**Mo26**	21	Negative	Negative	Negative	Negative
**Mo27**	21	Negative	Negative	Negative	Negative
**Mo28**	21	Negative	Negative	Negative	Negative
**Mo1**	21	Negative	Negative	Negative	Negative
**Mo2**	21	Negative	Negative	Negative	Negative
**Mo3**	21	Negative	Negative	Negative	Negative

Clinical Score Sheet for mice inoculated with tick-borne encephalitis virus (TBEV)

Table modified according to EC.Europe Directive 2010/63/EU (2012); (Foltz et al. 1999; OECD 2000; Pope et al. 2013; Morton and Griffiths, 1985; Visser, 1995; Conti et al., 2006; Haskins, 1992; Gärtner und Militzer, 1993; Becker, 2017)

Clinical score parameter severity of disorder in mice		Score
**Bodyweight**		
<5% weight loss		0
5-10% weight loss		1
11-20 % weight loss		2
acute weight loss > 20% in comparison to inital weight (≤24h)	*	HEP
weight loss > 25% in comparison to initial weight	*	HEP
**Cardiovascular system**		
normal		0
pinched skin/mild enophthalmia (sunken eyes)/mild dehydration		1
moderate to severe dehydration/moderate to severe ophtalmia (sunken eyes)		HEP
animal cold, legs and abdominal skin dark blueish		HEP
**Coat/Skin Condition**		
trimmed		0
Coat slightly unkempt, mild small skin irritation		1
Slight piloerection or small lesions, moderate small skin irritation or inflammation		2
Marked piloerection or moderate (scale) lesions or skin inflammation		3
Severe/large scale lesions, severe skin inflammation		HEP
**Respiratory tract**		
normal		0
Tachypnoea (slight)		1
Tachypnoea (moderate)		2
Dyspnoea (marked) <24h		3
Dyspnoea (marked) >24h		HEP
mild serous ocular or nasal discharge		1
moderate serous to purulent ocular or nasal discharge		2
severe serous to purulent ocular or nasal discharge		3
**Environment**		
normal		0
Loose stools or diarrhoea		1
haemorrhagic diarrhoea		HEP
**Social Behaviour/General condition/Locomotion**		
normal		0
Lack of grooming, mildly inactive (tired)		1
Not restricted in mobility, beginning kyphosis (hunched back), mildly inactive and depressed; Reduced interaction with other animals/apathic		2
hunched up back (kyphosis), lethargic, isolation or hyperactive, vocalization on moving		3
severe kyphosis		HEP
Immobility/moribund		HEP
**Neurological scoring**		
normal		0
mild neurological signs (e.g. head tilt, beginning ataxia, paralysis)		3
moderate to severe neurological signs, ataxic, paralysis, shaking, convulsions, moving in circles		HEP
**Other**		

**Actions**	
**CS 1**	Review frequency of monitoring
**CS 2**	≤ 12 h frequency of monitoring
**CS 4**	consult veterinarian
**CS 6 >48 h**	Implement humane endpoint
**CS=3 in >2 categories (highlighted in grey)**	Implement humane endpoint
**HEP**	Implement humane endpoint

*Im Folgenden werden die Abbruchkriterien aufgrund des Gewichtes im Detail erläutert:

1. „acute weight loss" > 20% in comparison to initial weight" = akuter Gewichtsverlust **>20% innerhalb 24 h** im Vergleich zum initialen Gewicht vor dem Versuch.

2. „weight loss > 25% in comparison to initial weight" = Gewichtsverlust **>25% im Vergleich zum initialen Gewicht vor dem Versuch (sofern Kriterium 1 „acute weight loss >20%" innerhalb 24 h nicht zutrifft)**.
   Das Allgemeinbefinden der Tiere sollte ansonsten ohne besonderen Befund sein (d.h. Clinical score in weiteren Kategorien = 0). Sollte das Tier einen Gewichtsverlust zwischen >20% und 25% haben und in anderen Kategorien einen Clinical Score von ≥1, wird das Tier unverzüglich euthanasiert

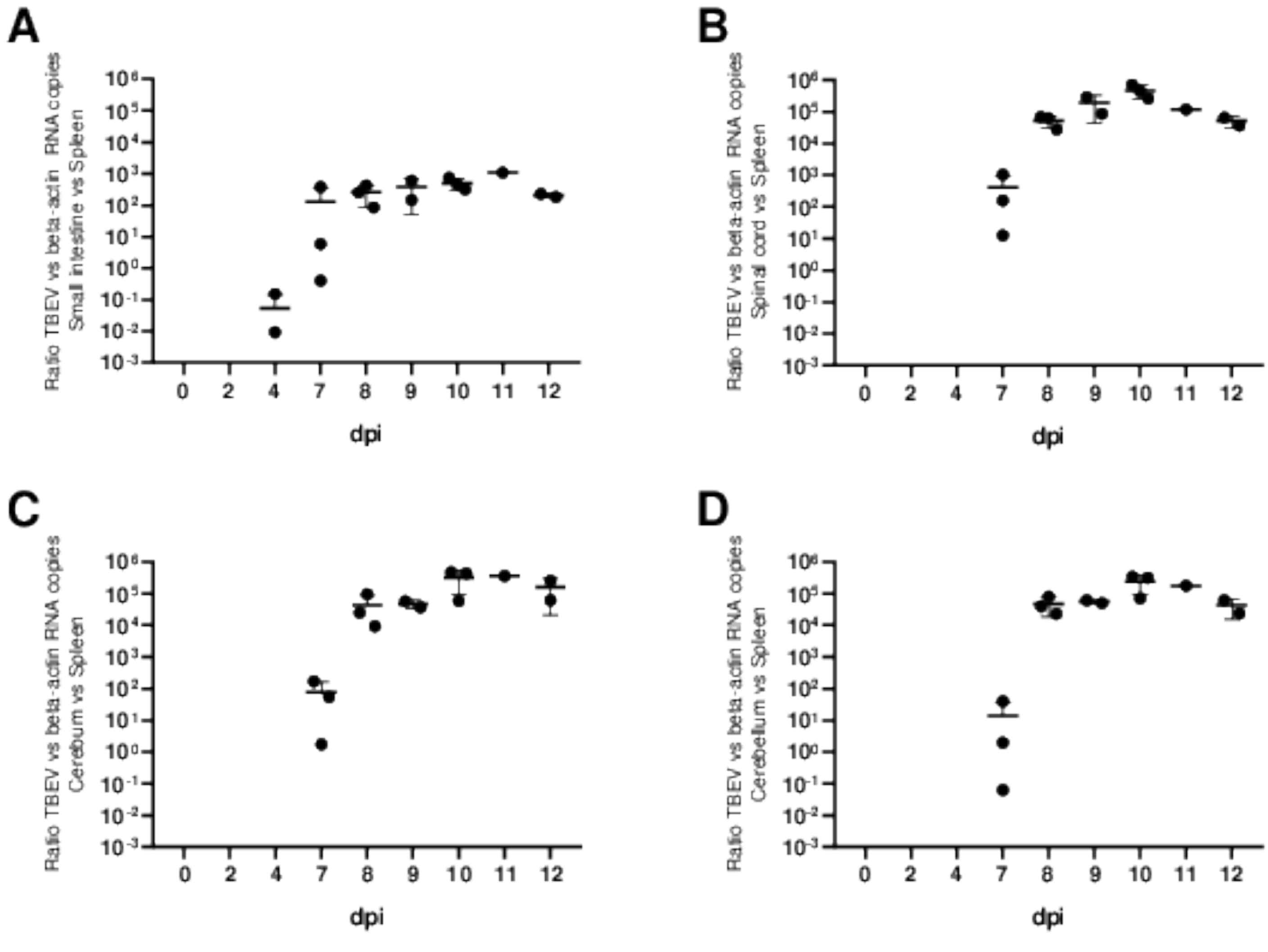
A
Ratio TBEV vs beta-actin RNA copies
Small intestine vs Spleen
dpi
B
Ratio TBEV vs beta-actin RNA copies
Spinal cord vs Spleen
dpi
C
Ratio TBEV vs beta-actin RNA copies
Cerebum vs Spleen
dpi
D
Ratio TBEV vs beta-actin RNA copies
Cerebellum vs Spleen
dpi
